# Self-Reported Academic Misconduct among Medical Students: Perception and Prevalence

**DOI:** 10.1155/2021/5580797

**Published:** 2021-08-23

**Authors:** Umar F. Dar, Yusuf S. Khan

**Affiliations:** ^1^Department of Community and Family Medicine, College of Medicine, Jouf University, Sakaka 72388, Al-Jouf, Saudi Arabia; ^2^Department of Basic Medical Sciences, College of Medicine, Vision (Alfarabi) Colleges, Riyadh 13226, Saudi Arabia

## Abstract

Academic integrity is the basis of an education system and must be taught as an ethical behavior during academic training. Students who reflect honesty and truthfulness during the academic years are more likely to follow this path, develop professional integrity, and thus become responsible and dependable professionals. Here, we determine the prevalence of academic lapses among medical students by a cross-sectional descriptive survey based on a self-assessment questionnaire. Students' perception of 37 behaviors comprising five domains, plagiarism, indolence, cheating, disruptive behavior, and falsifying data, were explored. A high percentage of students (83%) indicated that all 37 behaviors constitute misconduct. Approximately 65% of students thought that their fellow students were involved in dishonest behaviors, and 34% answered that they were indulged in some form of misconduct. Content analysis identified some prevalent behaviors such as doing work for another student (82.5%), getting information from the students who already took the exam (82.5%), copying the answer from neighbors (79%), and marking attendance for absent friends (74.5%). Multiple regression analysis points out that future indulgence in a behavior is significantly (*p* ≤ 0.5) correlated with understanding a behavior as wrong, perceiving that others do it and whether one has already indulged in it. This study can serve as a diagnostic tool to analyze the prevalence of misconduct and a foothold to develop the medical school system's ethical guidelines.

## 1. Introduction

The medical profession is regarded as the epitome of integrity and humanity. However, with commercialization and profitability seeping into the society, academic and professional misconduct behaviors have soared in developing countries. This is evident by reports of unethical practices, incompetence, and lack of responsibility in the medical profession [1, 2]. Quite a few studies report the prevalence of one or other kinds of academic misconduct in medical colleges worldwide [2, 3]. Some survey studies have observed that academic misconduct is lower in developed countries [4], while in developing countries, it can range from 50 to 99% [5]. This variation could be due to the cultural and socioeconomic disparity of different countries [6, 7] and a difference in the perception of misconduct [8, 9].

Medical professionals are desired to be competent and adhering to moral practices. Such qualities as professionals are underpinned by the stringency of academic training, development of understanding, and practice. To ensure ethics and prevent lapses, it becomes mandatory to know the most common causes of academic misconduct [10]. Primarily, it is observed that the lack of awareness of specific misconduct, i.e., plagiarism, among students [11], leads to indulgence in such practices. Notably, increasing awareness is shown to reduce the incidences [6, 12–14]. This study focuses on understanding the cause of academic misconduct, which may help in devising appropriate correction methodology to avoid or amend behavior discords [15].

Pakistan is a developing country with eastern culture and vast divisions in financial, social, and educational backgrounds [16]. Culturally, there is an inclination to choose the medical field because it is considered more prestigious than other professions. Hence, some students tend to employ unfair means to enter and succeed in this professional curriculum [17]. Reports point out that doctors trained in Pakistan and some other developing countries are more likely to be subjected to disciplinary actions by the medical councils than those trained in the developed countries [18, 19]. Academic misconduct thus indicates a serious issue affecting academic integrity in Pakistan, warranting identification and amendment. In this study, we choose Gujranwala Medical College (GMC) because the institute includes a diverse student population from various geographic areas who have obtained their preprofessional training from private and public sector institutions. We evaluated 37 types of academic misconduct and assessed how the perception of a particular behavior affects its prevalence. We also carried out correlation analyses to understand the relatedness of different misconduct behaviors to each other.

## 2. Materials and Methods

### 2.1. Study Design

A cross-sectional survey-based study was conducted at GMC, Pakistan. A total of 384 students, both male and female, from all levels of medical school were approached randomly for this study. A questionnaire consisting of 37 questions was given to each student, and they had the choice to participate anonymously. The questionnaire had an introductory page, explicitly stating that the data were collected for research purposes only. The study was commenced after the approval of the Research and Ethics Committee. Completed questionnaires were received only from 200 students (aged 22–28 years), spanning all academic years of the medical college.

### 2.2. Measurement Tools

Dundee Polyprofessional Inventory-1 customized for Pakistani medical colleges was used [20]. This included a validated scenario-based questionnaire divided into five academic misconduct domains, namely, plagiarism, indolence, cheating, disruptive behavior, and falsifying data.

### 2.3. Data Collection

The students were given the questionnaire, and the purpose of the study was clearly detailed to them. They also had the option to refuse or not to complete the questionnaires. Dundee Polyprofessional Inventory-1 was given as a paper questionnaire with a “yes and no” option against 37 behaviors, which were evaluated for each of the following four questions:

 
*Q1*. Is this wrong? (Evaluating perception)
 
*Q2*. Do you think fellow students do this? (Evaluating perceived prevalence)
 
*Q3*. Have you ever carried out this in your present course? (Evaluating prevalence)
 
*Q4*. Would you ever do this in your present course? (Evaluating possible future indulgence)

The 37 behaviors were segregated into the five domains that included plagiarism, indolence, cheating, disruptive behavior, and falsifying data (Table 1).

### 2.4. Data Analysis

The data were analyzed using SPSS version 22.0 (SPSS Inc., Chicago, Illinois, USA). Continuous variables are presented as mean ± standard deviation (SD), and categorical variables are presented as absolute and relative frequencies. Multiple linear regression analysis and Pearson's correlation were carried out using SPSS and cross-validated using NCSS software (NCSS 11 Statistical Software, 2016, NCSS, LLC. Kaysville, Utah, USA). The correlation and prevalence rate was calculated for each behavior to investigate the association between perceiving a dishonest behavior as wrong (Q1) and the future prospect of doing the same (Q4). A correlation was also generated between perceived prevalence (Q2) and actual prevalence (Q3) to understand a possible link between the two.

## 3. Results

Of 384 students invited to participate and approached for the study, 200 students completed the questionnaire. The participation percentage was 52.08%. There were 120 male responders and 80 female responders. The mean age (mean ± SD) of the participants was 25 ± 2.7 years. Table 1 specifies the number and percentage of the students answering the four questions (Q1–Q4) in yes or no format.

### 3.1. Perception (Q1)

The general perception of the students about wrong behaviors, spanning the five domains, was assessed through Q1. An average of 83% of the participants indicated that all 37 behaviors represent academic misconduct. Of 37 behaviors, 35 behaviors were perceived as wrong by more than 50% of the study population. Fourteen behaviors including using personal relationships or bribes to get academic advantages; exchanging answers using a mobile phone during the exam; taking unauthorized material in the exam; sitting in the exam for someone else or someone else sitting in the exam instead of the examinee; removing the reference from library shelf to prevent others from gaining access; damaging others work; drug abuse; posting inappropriate material about teachers and students in social media; and falsifying grades on CV were perceived as wrong by more than 90% of the study population.

Furthermore, 12 of 37 behaviors that include doing the work for another student, taking the ideas from someone and using as one's own, lack of punctuality for classes, altering data to get desired results, citing sources not fully read, damaging public property, forging healthcare worker's signatures, making false logbook entries, and presenting fraudulent certificates were identified as wrong behaviors by more than 80% of the students.

### 3.2. Perceived Prevalence (Q2)

The question “Do you think fellow students do this?” (Table 1) was aimed to assess whether the students perceive that the fellow students indulge in dishonest behaviors, spanning the five domains. In all the 37 behaviors, the perceived prevalence (65%) was consistently higher than the individual indulgence in fraudulent behavior (Q3). Few prominent observations for perceived prevalence were removing a reference from a library shelf (38% compared to 2% prevalence), deliberately damaging another student's work (35% compared to 4%), taking unauthorized material to the exam (66.5% compared to 10.5%), examining the patient without the supervisor's consent (76.5% compared to 22.5%), and forging healthcare worker's signature (60.5% compared to 19% prevalence).

Notably, we observed that the perceived prevalence was very high in the disruptive behavior domain compared to the other four domains. Abusing (59.5%) and physically assaulting (58.5%) a university employee or student, damaging public property (72%), controversial social media content about teachers and students (55%), and inappropriate medicine presentation in social media (33%) are behaviors in the domain of disruptive behavior with significantly higher perceived prevalence (Q2) than the actual prevalence (Q3).

### 3.3. Prevalence (Q3)

We observed that some behaviors are more prevalent than others. In the five domains presented in our questionnaire, behaviors in the cheating domain are the most common among students. As indicated in Table 1 (Q3), 82.5% of the students admitted that they had carried out work for another student and an equal number of students accepted getting information from fellow students who have already taken the exam. It is also observed that 79% mentioned copying answers from the neighbors and 71% admitted to passing the information to other students who would take the exam. Copying the text from the source was also prevalent with 51.5% of the study population.

Other moderately prevalent cheating behaviors are giving help for coursework against the rule (54.5%), taking the idea for work from fellow students and claiming it as their own (49.5%), copying the text directly (51.5%), citing the source not thoroughly read (44.5%), and altering the data to get desired results (53.5%). In the domain falsifying the data, 74.5% of students admitted that they mark attendance for absent friends and 35% indicated that they make false entries in logbooks.

### 3.4. Possible Future Indulgence (Q4)

The possibility of a person indulging in a wrong behavior was assessed by Q4. The percentage of “yes” answers for this question was generally low (ranging between 2 and 39%), with an average of 21.2%. However, a high rate of “yes” responses for future indulgence was also observed for a few types of misconduct. In all, 71% of the participants admitted that they would get information from fellow students who have already sat in the exams, 66% indicated copying answers from the neighbors during the exam, 65.5% indicated that they would pass the information to the students who have to take the exam, and 65% indicated that they would mark the attendance for absent fellow students. A significant percentage of the participants (57%) also indicated that they would do the work for another student, and 56.6% of participants indicated they would photograph cadavers and dissected materials.

### 3.5. Correlation between Perception, Prevalence, Perceived Prevalence, and Future Indulgence in Misconduct

Pearson correlation (Table 2) was calculated for the response of the participants to the four questions (Q1–Q4) to identify how perception, prevalence, perceived prevalence, and the possibility of future indulgence in a wrong behavior are connected. A correlation was derived between Q1 (perception) and Q4 (future indulgence), Q1 (perception) and Q3 (prevalence), and Q2 (perceived prevalence) and Q3 (prevalence). A very strong negative correlation was demonstrated for all five domains between Q1 and Q4, namely, plagiarism and indolence (*R* = −0.9751), cheating (*R* = −0.8424), disruptive behavior (*R* = −0.976), and falsifying data (*R* = 0.98593). The correlation analysis between Q1 (perception) and Q3 (prevalence) also demonstrated a significant negative correlation for three domains, i.e., cheating (*R* = −0.8143), disruptive behavior (*R* = −0.928), and falsifying data (*R* = −0.9622). However, a significantly moderate positive correlation between Q2 (perceived prevalence) and Q3 (prevalence) was observed for plagiarism and indolence (*R* = 0.8242), cheating (*R* = 0.8234), and falsifying data (*R* = 0.8514).

A negative correlation between Q1 and Q4 for the behaviors of the five domains points out that identifying a behavior as wrong could potentially prevent a person from doing so in the future. Similarly, a negative correlation between Q1 and Q3 for behaviors in the three domains indicates that if individuals perceive a behavior as wrong, there is a high possibility of them not doing it. A moderate positive correlation between Q2 and Q3 indicates that perceived prevalence according to the participants actually may correspond to prevalent misconduct in medical schools.

### 3.6. Effect of Perception and Prevalence on Future Indulgence in Misconduct

Multiple linear regression analysis (Table 3) was carried out to identify the interaction between the dependent variable, Q4, which determines an individual's possibility of indulging in a wrong behavior in the future, and dependent variables, Q1 (perception), Q2 (perceived prevalence), or Q3 (prevalence) for each of the domain. A significant interaction was obtained for all domains, namely, plagiarism and indolence (*p* < 0.05), cheating (*p* < 0.001), disruptive behavior (*p* < 0.001), and falsifying data (*p* < 0.001). Hence, the findings of this study reveal a significant relationship between future indulgence to perception, prevalence, or perceived prevalence.

This regression analysis accounted for 88.48% of the variance, with adjusted *R* square = 0.08 (95% CI). Overall, the results indicate that perception, perceived prevalence, or prevalence of a wrong behavior are significant predictors of future indulgence in academic misconduct in all five categories.

## 4. Discussion

The present study was carried out to investigate the attitude of academic misconduct among undergraduate medical students and how their perception of a particular behavior is related to its prevalence and possible future indulgence in misconduct. The study was carried out in GMC, Pakistan, which enrolls students from diverse educational backgrounds. The ethical training imparted to the students prior to entering medical school and during the first year of medical school shapes their perception of misconduct. Students entering GMC have completed their secondary school education from institutions following international boards such as the British curriculum as well as local/regional boards. Hence, GMC provided us with a varied background of moral understanding of the participants during their secondary and senior secondary school training. It is recognized that most institutions have an ethics course taught as a part of the curriculum. GMC also has a medical ethics course during the first semester of the curriculum underpinning that the students enrolled in the study have an understanding of medical ethics.

In this study, we observed that the incidence of lapses in academic integrity was significantly higher than in several studies conducted in developed countries [21–23]. Herein, the participants admitted indulging in most of the 37 behaviors listed in the Dundee Polyprofessional Inventory-1 (Table 1). Overall, the perceived prevalence was more than the actual indulgence in most behaviors (Table 1). A similar study conducted on participants from 31 schools also demonstrated that the actual prevalence of cheating was 4.7%, while the perceived prevalence was 39%, similar to our study [24]. At the same time, some other studies indicate a substantial correlation between perception and prevalence [25, 26].

A high prevalence of academic misconduct in some countries may be related to cultural and socioeconomic backgrounds [6, 18]. In some societies, misconduct behaviors such as ‘doing the work for fellow students' or ‘ giving help to fellow students for assignments' are understood as co-operation, support, and benevolence [27–29]. In few developing countries, students sometimes depend on using unethical means to achieve their target, mostly when they are incapable of properly carrying out an assigned task [30]. Nonetheless, a significant negative correlation between Q1 and Q4 in all four domains of our study (Table 2) indicates that understanding a behavior as wrong could psychologically influence a person to refrain from doing it [31, 32]. Similarly, a moderate positive correlation observed between Q2 and Q3 indicates that observing a wrong behavior may potentially desensitize a person towards the action and hence could propel them to indulge in it (Table 3 (A, B)). Students witnessing a fellow student succeed by cheating may influence them to adopt cheating. Therefore, we conclude that the perception of behavior potentially affects its prevalence [31].

Misconduct behaviors such as plagiarism and falsifying data (Table 1) could result from ignorance, probably due to lack of information or setting unrealistic goals focusing only on being successful or getting higher grades [33, 34]. Shirazi et al. surveyed three medical institutes in Karachi, Pakistan, and found a general lack of information about plagiarism among the students [35]. Studies indicate that majority of the students did not have the proper knowledge about appropriate referencing and needed more training to prevent plagiarism [36–39]. A poor understanding of the English language among some students also plays a significant part in cheating and plagiarizing. Hence, students find writing and communication in the English language difficult and may choose to copy from different sources like the Internet and others.

Indolence, such as lack of punctuality and failing to follow standards in educational settings, sometimes stems from the culture in addition to the individual's responsible attitude [40]. Lack of timeliness and irregularity in working towards deadlines are generally reflected in the Pakistani society as an acceptable social norm. Furthermore, some academic misconduct results from emotional outbursts that can disrupt the usual demeanor of a person. Disruptive behaviors that inflict physical or moral insult to others may result from a person's inability to control themselves. However, some actions such as deliberately damaging a fellow person's work or delaying exams may be due to pressure to succeed academically or fear of losing status with peers [41, 42].

Unprofessional behavior during academic life is considered a substantial predictor of disciplinary action during professional life [43, 44]. Ethical lapses are usually carried over to one's profession and tend to affect and influence the people in such spheres. Hence, an approach to address different ethical components of the curriculum needs to be structured and implemented in the Pakistani society [45, 46]. Studies have proved that when the students are made aware of the constituents of plagiarism and its ill effects, their indulgence in such practices is observed to decline substantially [47–49]. It has also been understood that high competition leading to stress, anxiety, and depression among students can result in plagiarism. Hence, training them to cope with stress, anxiety, and depression and improving their mental health, in turn, can help reduce academic misconduct [50, 51]. To publicize a research misconduct policy (RMP) in each university describing the types of misconduct, inquiry, investigation, and strict penalties on breaching the same might also help in shrinking the number and better handling of the cases [52].

## 5. Conclusions

Many factors influence misconduct in an academic setting. In this study, we have assessed 37 behaviors that constitute misconduct prevalence in the medical education environment. We have linked the role of perception about wrong behavior and its perceived prevalence and actual prevalence to future indulgence. Our results demonstrate that individual perception about a wrong behavior and its prevalence in an academic setting can significantly predict if an individual is prone to future indulgence. This study can serve as a diagnostic tool to understand the level of misconduct present in medical colleges. Further research through the enrolment of students from medical colleges across the country to analyze the influence of socioeconomic status, religiosity, and gender differences on academic integrity may provide a more in-depth understanding of the factors that lead to misconduct.

## Figures and Tables

**Table 1:** Self-reported behavior of medical students regarding academic dishonesty.

S. no.	Behaviours	Q1: perception Is this wrong? *N* (%)	Q2: perceived prevalence Do you think fellow students do this? *N* (%)	Q3: prevalence Have you ever done this in the present course? *N* (%)	Q4: future indulgence Would you do this in the present course? *N* (%)	Type of misconduct
1	Take the idea or work from a fellow student and pass it as one's own	171 (85.5)	158 (79)	99 (49.5)	29 (14.5)	P
2	Resubmitting work already submitted for another assignment	163 (81.5)	134 (67)	79 (39.5)	38 (19)	P
3	Copying the text directly from a source	153 (76.5)	165 (82.5)	103 (51.5)	58 (29)	P
4	Missing lectures frequently	170 (85)	172 (86)	90 (45)	63 (31.5)	I
5	Failing to follow the standard infection control protocols	172 (86)	135 (67.5)	48 (24)	20 (10)	I
6	Lack of punctuality for classes	177 (88.5)	164 (82)	83 (41.5)	40 (20)	I
7	Photographing cadavers or dissected materials	124 (62)	167 (83.5)	91 (45.5)	53 (56.5)	I
8	Altering the data to get the desired result	171 (85.5)	157 (78.5)	107 (53.5)	74 (37)	C
9	Doing the work for another student	91 (45.5)	167 (83.5)	165 (82.5)	114 (57)	C
10	Giving help for coursework against the rule	157 (78.5)	167 (83.5)	109 (54.5)	60 (30)	C
11	Claiming teamwork as individual work	175 (87.5)	136 (68)	77 (38.5)	25 (12.5)	C
12	Paying a fellow student for completion of course work	132 (66)	80 (40)	53 (26.5)	39 (19.5)	C
13	Citing the sources not fully read	172 (86)	147 (73.5)	89 (44.5)	39 (19.5)	C
14	Accessing the papers which have not been released to the whole class	174 (87)	137 (68.5)	62 (31)	39 (19.5)	C
15	Using personal relationships or bribes to get an academic advantage	181 (90.5)	139 (69.5)	58 (29)	43 (21.5)	C
16	Copying answers from a neighbor during exams	136 (68)	180 (90)	158 (79)	132 (66)	C
17	Exchanging answers using mobile phones during exams	184 (92)	144 (72)	65 (32.5)	16 (8)	C
18	Getting information about the exam from students who have already taken the exam	119 (59.5)	184 (92)	165 (82.5)	142 (71)	C
19	Passing information about the exam to students who have to take the exam	113 (56.5)	177 (88.5)	143 (71.5)	131 (65.5)	C
20	Taking unauthorized materials in the exam	187 (93.5)	133 (66.5)	21 (10.5)	10 (5)	C
21	Sitting in the exam for someone else or someone else sitting in the exam for you	191 (95.5)	96 (28)	13 (6.5)	4 (2)	C
22	Removing a reference from the library shelf to prevent other students from gaining access to the information	188 (94)	76 (38)	4 (2)	12 (6)	D
23	Deliberately damaging another student's work	185 (92.5)	70 (35)	8 (4)	7 (3.5)	D
24	Creating circumstances to delay the exams	151 (75.5)	139 (69.5)	90 (45)	78 (39)	D
25	Abusing a university employee or a student	181 (90.5)	119 (59.5)	32 (16)	11 (5.5)	D
26	Physically assaulting a university employee or a student	184 (92)	117 (58.5)	18 (9)	15 (7.5)	D
27	Drug abuse	184 (92)	126 (63)	16 (8)	7 (3.5)	D
28	Providing illegal drugs to the students	186 (93)	84 (42)	38 (19)	11 (5.5)	D
29	Damaging public property	178 (89)	144 (72)	29 (14.5)	24 (12)	D
30	Inappropriate materials about students or teachers on social media	184 (92)	111 (55.5)	16 (8)	8 (4)	D
31	Inappropriate presentation of medicine on social media	181 (90.5)	66 (33)	22 (11)	13 (6.5)	D
32	Marking attendance sheet for absent friends	143 (71.5)	181 (90.5)	149 (74.5)	130 (65)	F
33	Examining the patients without the consent of the supervisor	180 (90)	153 (76.5)	45 (22.5)	17 (8.5)	F
34	Forging a health care worker's signature	175 (87.5)	121 (60.5)	38 (19)	18 (9)	F
35	Falsifying grades on CV or treatment sheets	185 (92.5)	58 (29)	6 (3)	9 (4.5)	F
36	Making false entries in logbooks	163 (81.5)	119 (59.5)	70 (35)	62 (31)	F
37	Presenting false certificates	177 (88.5)	63 (31.5)	12 (6)	16 (8)	F

Average “yes” answers (%)		83.0	65.0	34.1	23.2	

The table lists the 37 behaviors of Dundee Polyprofessional Inventory-1 customized for Pakistani medical colleges relating to academic misconduct. Four questions, Q1–Q4, were asked to the participants in yes/no format. The data presented here indicate the number of “yes” answers for each question. *N* represents the number, and % represents the percentage of participants of total (*N* = 200) answering as “yes.” The types of misconduct are represented by plagiarism (P), indolence (I), cheating (C), disruptive behavior (D), and falsifying data (F).

**Table 2:** Correlation between Q1 and Q4, Q1 and Q3, and Q2 and Q3.

		Correlation between Q1 and Q4	*p* value	Correlation between Q1 and Q3	*p* value	Correlation between Q2 and Q3	*p* value
1	Plagiarism and indolence	−0.8865	<0.01	−0.3169	<0.5	0.8242	<0.05
2	Cheating	−0.8424	<0.001	−0.8143	<0.001	0.8234	<0.001
3	Disruptive behavior	−0.9762	<0.001	−0.928	<0.001	0.5011	<0.1
4	Falsifying data	−0.9859	<0.05	−0.9622	<0.01	0.8514	<0.05

The correlation was obtained using Pearson's correlation. The *p* value indicates the significance of the correlation between the questions (Q). The *p* value of ≤0.01 was considered significant.

**Table 3:** Multiple linear regression analyses to assess the effect of Q1, Q2, and Q3 on Q4 (future indulgence in a behavior).

A.			
		Influence of Q1, Q2, and Q3 on Q4 (future indulgence)	
		*R* ^2^	*p* value

1	Plagiarism and indolence	0.9424	<0.05
2	Cheating	0.9544	<0.001
3	Disruptive behavior	0.9055	<0.001
4	Falsifying data	0.9987	<0.001

B.			
		Influence of Q1, Q2, and Q3 on Q4 (future indulgence): *β*	
		Variables	Beta (*β*)

1	Plagiarism and indolence	Q1	−1.3692
		Q2	1.0357
		Q3	−0.3216

2	Cheating	Q1	−0.3616
		Q2	0.0366
		Q3	0.6873

3	Disruptive behavior	Q1	−1.8825
		Q2	−0.0259
		Q3	0.0575

4	Falsifying data	Q1	0.0401
		Q2	−0.447
		Q3	1.2391

The impact of three independent variables (Q1, Q2, and Q3) was obtained to predict their effect on the dependent variable (Q4). The correlation between predictors is significant and ranges between ≤0.05–<0.001. The high scores for R2 in A indicate a strong correlation and a pronounced effect of the three variables on future indulgence. B shows that all three variables contribute significantly to the future indulgence.

## Data Availability

The data used to support this study are included within the article.
